# Ellagic Acid Attenuates High-Fat, High-Sucrose Diet-Induced Cardiac Fibrosis by Suppressing AGE–RAGE Signaling, Oxidative Stress, and Inflammation

**DOI:** 10.3390/ijms27198658

**Published:** 2026-09-28

**Authors:** Soumya R. Gurrala, Dileep R. Rajapuram, Prathap R. Kallamadi, Sneha Jakhotia, Vandana V. Sharma, Parakh P. Mody, Samarasimha N. Reddy, G. Bhanuprakash Reddy

**Affiliations:** 1 Biochemistry Division, ICMR-National Institute of Nutrition, Hyderabad 500007, India; soumyareddygurrala1701@gmail.com (S.R.G.); vandana13203@gmail.com (V.V.S.); parakh.mody@icmr.gov.in (P.P.M.); 2 Clinical Epidemiology, ICMR-National Institute of Nutrition, Hyderabad 500007, India; reddy.samarasimha@icmr.gov.in

**Keywords:** advanced glycation end products (AGEs), extracellular matrix (ECM), cardiac fibrosis, ellagic acid, inflammation, nuclear factor kappa-light-chain-enhancer of activated B cells (NF-κB)

## Abstract

A high-fat, high-sucrose (HFHS) diet promotes insulin resistance and accelerates cardiac fibrosis (CF) through advanced glycation end products (AGEs), oxidative stress, and inflammation. Ellagic acid (EA) possesses antioxidant, anti-inflammatory, and anti-glycation properties; however, its protective effects against CF and underlying molecular mechanisms remain unclear. Therefore, we investigated EA’s effects on HFHS-induced CF. Two-month-old male Wistar rats were randomly assigned to four groups (n = 6/group): control, control + 2% EA, HFHS, and HFHS + 2% EA, and maintained on respective diets for six months. Metabolic parameters, oxidative stress, AGE-related markers, histopathology, immunoblotting, and immunohistological analyses were performed. HFHS feeding induced metabolic dysfunction characterized by weight gain, increased fasting blood glucose levels, insulin resistance, dyslipidemia, myocardial disorganization, AGE accumulation, receptor for AGE (RAGE) activation, collagen deposition, and fibrotic marker expression- alpha smooth muscle actin (α-SMA). EA supplementation significantly improved metabolic abnormalities, reduced myocardial collagen fraction, AGE accumulation, RAGE activation, and α-SMA expression, thereby protecting against HFHS-induced CF.

## 1. Introduction

Cardiovascular diseases (CVDs) are the leading cause of mortality and disability within the Association of Southeast Asian Nations (ASEAN) region, with ischemic heart disease and peripheral arterial disease significantly impacting the overall burden [1]. Metabolic and lifestyle factors such as unhealthy dietary patterns, obesity, insulin resistance, hyperglycemia, and dyslipidemia play a role in the development of CVDs [2]. Experimental studies demonstrate that high-fat and high-carbohydrate diets promote metabolic dysfunction, insulin resistance, and adverse cardiovascular remodeling [3,4]. Cardiac fibrosis (CF) is a key pathological outcome of cardiac remodeling, marked by the excessive accumulation of extracellular matrix (ECM) proteins, resulting in increased myocardial stiffness and impaired cardiac function [5]. The ECM is a dynamic network of proteins that helps maintain the structural integrity of tissues and plays a crucial role in regulating fibroblast behavior in both healthy and disease states [6]. Persistent metabolic stress disrupts ECM homeostasis, activates fibroblasts, and leads to increased collagen deposition, thus contributing to progressive cardiac remodeling and dysfunction [7].

A key mechanism linking metabolic stress to fibrotic remodeling is the accumulation of advanced glycation end products (AGEs). Higher glucose levels and oxidative stress accelerate the formation and accumulation of AGEs on long-lived proteins, including ECM components such as collagens and laminins [8,9]. AGEs induce crosslinking between these matrix proteins, thereby stiffening the ECM and reducing tissue/myocardial compliance [8,10]. In addition to these structural effects, binding of AGEs to their receptor RAGE (receptor for AGEs) and subsequent activation of the AGE/RAGE axis stimulates fibroblast proliferation and myofibroblast differentiation, inducing oxidative stress and triggering inflammatory responses [11,12]. Experimental studies in primary cardiac fibroblasts have further shown that AGEs activate intracellular signaling and ECM-remodeling pathways associated with pathological cardiac remodeling [13].

Moreover, we recently reported that AGE adduct formation on ECM augments fibroblast activation and contributes to fibrosis [6]. Experimental evidence also demonstrates that AGE-RAGE signaling can contribute to CVD through interconnected mechanisms involving oxidative stress, inflammation, and tissue fibrosis [14,15]. Altogether, these findings support that the AGE/RAGE axis is an important mechanistic link between chronic metabolic stress, oxidative stress, inflammation, fibroblast activation, and ECM remodeling.

Ellagic acid (EA), a naturally occurring polyphenolic compound present in numerous fruits and vegetables, possesses antioxidant, anti-inflammatory, and anti-glycating properties [16,17]. EA has demonstrated the ability to prevent the formation of AGEs and modifications of glycation-related proteins, highlighting its potential to reduce molecular damage associated with AGEs [17]. In streptozotocin-induced rats, EA preserved cardiac architecture, reduced collagen deposition, and modulated SIRT1/TGF-β1/Smad3 signaling, implicating its protective effects [18]. EA has also been reported to suppress cardiac fibroblast proliferation and migration and reduce collagen expression by downregulating HDAC1 [19]. More recently, EA was shown to attenuate cardiac injury by reducing oxidative stress and ferroptosis through modulation of the SIRT1/p53 pathway [20]. In addition, ex vivo studies using isolated and perfused rat hearts demonstrated that EA can directly influence myocardial contractility and calcium handling, further supporting its biological activity in cardiac tissue [21]. Collectively, these studies indicate that EA can modulate the processes relevant to cardiac remodeling, including oxidative stress, inflammation, fibroblast activation, ECM deposition, and cellular stress-related signaling.

Despite these findings, most previous studies have focused on chemically induced models, isolated cardiac preparations, cardiac fibroblast systems, or specific molecular pathways. Consequently, the effects of EA under prolonged HFHS-diet-induced metabolic stress remain less characterized. Comprehensive studies integrating glycation markers, oxidative stress, inflammation, and ECM remodeling in the HFHS-induced model are limited. Therefore, in the present study, we investigated the protective effects of EA on cardiac fibrosis in a high-fat, high-sucrose (HFHS) diet-fed rat model, with a particular focus on its ability to modulate AGE formation, AGE/RAGE signaling together with oxidative stress, and inflammation, thereby addressing a gap in the current understanding of EA in HFHS-associated cardiac fibrotic remodeling.

## 2. Results

### 2.1. Effect of Ellagic Acid on Metabolic Parameters

Although food intake did not differ significantly from the control group (Figure 1A), rats fed the HFHS diet for six months had considerably increased body weight. However, EA supplementation did not affect weight gain (Figure 1B). To assess glucose metabolism, an oral glucose tolerance test (OGTT) was conducted at the end of the experiment. Compared with the control group, the HFHS group had higher fasting blood glucose (FBG) levels (Figure 1C) and impaired glucose clearance after oral glucose administration (Figure 1D). Additionally, the HFHS group had significantly higher fasting insulin levels and a marginal increase in HOMA-IR, suggesting possible insulin resistance due to HFHS feeding (Figure 1E,F). However, EA supplementation improved insulin sensitivity, indicated by decreased blood glucose, area under the curve (AUC) calculated from OGTT, insulin levels, and HOMA-IR (Figure 1C–F).

### 2.2. Effect of Ellagic Acid on Body Composition and Lipid Profile

Since HFHS feeding resulted in increased insulin levels, we further evaluated lipid profile and body composition to assess the associated metabolic alterations. The HFHS diet-fed group had higher total cholesterol and triglyceride levels, whereas EA supplementation significantly improved the lipid profile (Figure 2A,B). Dual-energy X-ray Absorptiometry (DEXA) reports show that although lean body mass decreased, the change was not statistically significant. However, fat mass differed significantly between the C+EA and HFHS groups (Figure 2C,D). EA supplementation did not significantly affect body composition (Figure 2C,D).

### 2.3. Effect of Ellagic Acid on HFHS-Induced Cardiac Histomorphological Changes

Hematoxylin and eosin (H&E) staining showed some disorganization of myocardial structure in the HFHS diet-fed group compared with the control group. However, EA treatment appeared to reduce these histomorphological changes (Figure 3A). Masson’s Trichrome (MT) staining of heart sections from the HFHS group showed increased collagen deposition in the interstitial and perivascular areas, indicating that the HFHS diet may lead to fibrotic changes (Figure 3B,C). EA supplementation significantly reduced collagen accumulation in both interstitial and perivascular areas (Figure 3B,C).

### 2.4. Ellagic Acid Prevents HFHS-Induced Fibrotic Changes

As we observed an increase in accumulation of collagen in the interstitial and perivascular spaces in the HFHS group (Figure 3B,C), we assessed two important ECM proteins linked to tissue remodeling and fibrosis- fibronectin, and collagen type 1 alpha (Col1α). Fibronectin expression was higher in the HFHS group (Figure 4A,B), whereas Col1α showed a modest, non-significant increase, suggesting that histological staining may capture broader collagen beyond Col1α (Figure 4C,D). Furthermore, α-smooth muscle actin (α-SMA), a hallmark of myofibroblast activation and fibroblast-to-myofibroblast transformation, was significantly increased in the HFHS group (Figure 5A,B). However, EA supplementation significantly decreased α-SMA expression, suggesting a protective effect against fibroblast activation.

### 2.5. Ellagic Acid Supplementation Ameliorates Protein Glycation Under HFHS Diet Conditions

Although EA did not affect plasma AGEs (Figure 6A,C), it significantly decreased AGE accumulation in the heart (Figure 6B). Plasma CML levels did not differ among groups. However, tissue Nε-(carboxymethyl) lysine (CML), a prominent non-fluorescent AGE, and methylglyoxal (MGO), a highly reactive glycation precursor, were higher in the HFHS group (Figure 6D–F). EA supplementation demonstrated its anti-glycation effect by lowering the levels of fluorescent AGEs, cardiac CML, and MGO. These results suggest that EA limits glycation-mediated tissue damage by guarding against AGE buildup induced by the HFHS diet.

### 2.6. Ellagic Acid Attenuates HFHS Diet-Induced Oxidative Stress and Inflammation

HFHS diet administration significantly increased plasma malondialdehyde (MDA) levels and protein carbonyls, indicative of oxidative modifications in circulating proteins and lipids (Figure 7A,B). Expression of NF-κB and its phosphorylated form (p-NF-κB) did not differ significantly between the heart tissue from control and HFHS groups. However, EA supplementation in HFHS-fed rats was associated with reduced NF-κB expression, suggesting a potential dampening effect on inflammatory signaling (Figure 7C,D). These findings suggest that EA supplementation may mitigate HFHS diet-induced metabolic alterations by lowering oxidative stress and attenuating NF-κB-driven inflammatory signaling pathways. RAGE expression was significantly higher in the HFHS group compared with the control group (Figure 7E,F), consistent with activation of the AGE/RAGE axis. In EA-supplemented rats, RAGE levels were lower than in HFHS rats, suggesting that EA may moderate this pathway, although differences between HFHS and HFHS+EA were not significant (Figure 7E,F).

## 3. Discussion

Metabolic dysfunction driven by insulin resistance is a rapidly growing global health concern and a major risk factor for cardiovascular complications [2]. Global burden studies confirm the rising prevalence of metabolic disease and its cardiovascular complications [1]. Dietary patterns rich in high-fat, high-sucrose (HFHS) components are implicated in the development of metabolic syndrome and can promote dyslipidemia, oxidative stress, inflammation, and accumulation of advanced glycation end products (AGEs) [3,4,7,8,9]. Importantly, these metabolic disturbances can contribute to cardiac remodeling in the presence of insulin resistance [7,10,22].

Natural polyphenolic compounds such as ellagic acid (EA) have attracted considerable attention due to their antioxidant, anti-inflammatory, and metabolic regulatory properties [16,17]. Furthermore, EA has been reported to inhibit and prevent AGE formation or accumulation, thereby mitigating metabolic complications [17].

The present study demonstrates that prolonged HFHS diet feeding induces metabolic abnormalities characteristic of insulin resistance, including impaired glucose homeostasis, dyslipidemia, increased adiposity, and reduced lean body mass. EA supplementation lowered fasting glucose and improved insulin sensitivity, suggesting a beneficial effect on basal glucose homeostasis, which is consistent with previous reports demonstrating that EA improves insulin signaling [23,24]. Additionally, the improvement in lipid profile following EA treatment further supports EA’s role in alleviating HFHS-induced metabolic disturbances. Interestingly, a study conducted in a type 2 diabetic model has reported beneficial effects of EA nanoparticles on lipid metabolism [25]. 

Oxidative stress and inflammation are recognized as important contributors to HFHS-induced cardiac remodeling [26]. The observed increase in methylglyoxal (MGO), Nε-(carboxymethyl)lysine (CML), and RAGE expression further suggests activation of the AGE-RAGE signaling axis under HFHS conditions, consistent with the reports linking AGE/RAGE signaling to NF-κB activation and fibrosis [12,27,28]. Other studies emphasize AGE cross-linking as an important mechanism of tissue stiffening and remodeling [10,22,29]. These mechanisms are not mutually exclusive, as AGE accumulation may contribute to cardiac injury through both structural modification of extracellular matrix proteins and receptor-mediated inflammatory signaling [10,22]. Further, AGE-RAGE interactions trigger downstream oxidative and inflammatory signaling pathways, including NF-κB activation [8,11,12]. In fact, a study showed that NF-κB deletion inhibited fibroblast-to-myofibroblast differentiation and reduced inflammatory cell recruitment, revealing NF-κB as an important mediator of cardiac remodeling [30].

In our study, HFHS-fed animals exhibited higher plasma malondialdehyde (MDA) levels and protein carbonyls, while EA supplementation lowered protein carbonyls and reduced NF-κB expression. Interestingly, EA supplementation attenuated these pathological alterations, as evidenced by reduced AGE accumulation,and collagen deposition in addition to decrease in both α-SMA and RAGE expression. These effects are consistent with reports that EA protects against cardiac stress, improves myocardial contractility [21], enhances mitochondrial function [31], reduces ROS generation, and attenuates cardiomyocyte apoptosis [32]. However, the present study evaluated only selected markers of oxidative damage; these findings should not be interpreted as a comprehensive assessment of oxidative stress. The beneficial effects of EA may involve both direct modulation of redox-related pathways and indirect effects associated with reduced AGE formation and AGE-RAGE signaling [31,33].

Collectively, our findings show that HFHS feeding induces myocardial remodeling characterized by disrupted myocardial architecture, collagen deposition, and increased fibrotic markers. EA supplementation significantly attenuated these pathological changes, supporting its potential to mitigate HFHS-induced metabolic dysfunction. The differences observed across studies likely reflect variations in EA dose, treatment duration, timing of intervention, and disease model, particularly the distinction between insulin-resistant metabolic syndrome and established diabetes [23,24,25]. Thus, the present findings extend previous observations by showing that EA can attenuate metabolic and cardiac remodeling associated with prolonged HFHS exposure, while highlighting the importance of disease context and treatment conditions when interpreting its cardiometabolic effects. Further comparative studies examining EA dose–response relationships and treatment timing across different stages of metabolic disease would help clarify when its benefits are most pronounced.

## 4. Materials and Methods

### 4.1. Chemicals and Reagents

Ellagic acid (ASB # 00005070-101, Chroma Dex, Irvine, CA, USA)**,** Tris-buffer, glycine, acrylamide, bis-acrylamide, ammonium persulfate, β-mercaptoethanol, sodium dodecyl sulfate, tetramethyl ethylenediamine, anti-mouse and anti-rabbit secondary antibodies, and 4,6-diamidino-2-phenylindole (DAPI) were purchased from Sigma-Aldrich (St. Louis, MO, USA). Alexa Fluor secondary antibodies and primary antibodies for methylglyoxal (MA5-27567), GAPDH (MA5-15738), RAGE (PA1-075), collagen 1 (PA5-29569), and fibronectin (PA5-29578) were obtained from Thermo Fisher Scientific (Waltham, MA, USA). The primary antibody for CML (ab27684) was purchased from Abcam (Cambridge, UK). NF-κB (8242s), p-NF-κB (3033s), and α-SMA (19245s) were purchased from Cell Signaling Technology (Danvers, MA, USA). The ELISA kit for rat insulin (10-1250-01) was procured from Mercodia (Uppsala, Sweden), and the rat CML ELISA kit (ITER0134) was procured from G-Biosciences (St. Louis, MO, USA). Lipid profile assay kits for plasma parameters were procured from Biosystems, Barcelona, Spain.

### 4.2. Animal Experimentation

Two-month-old male Wistar rats (294 ± 4 g) were procured from the Animal Facility, ICMR-National Institute of Nutrition (ICMR-NIN), Hyderabad, India. Animals were housed individually under controlled environmental conditions (22–24 °C, 50–60% relative humidity, and a 12 h light/dark cycle) with ad libitum access to food and water. Following a one-week acclimatization period, the animals were randomly assigned to four experimental groups with six animals in each group. The control diet (C), control diet supplemented with 2% EA (C+EA), high-fat,high-sucrose diet (HFHS; HF-45% and HS-20.5%) and HFHS diet supplemented with 2% EA (HFHS+EA) were prepared. The HFHS diet was prepared as reported previously [34]. The diets supplemented with 2% EA were prepared based on the dosage used in our previous study [35]. The experimental diets were administered for six months. Daily food intake and weekly body weights were monitored throughout the study. Fasting blood glucose was assessed periodically during the study.

At the end of the intervention period, following an overnight fast, animals were euthanized by CO_2_ asphyxiation. Blood samples were collected from all six rats in each group (n = 6/group), plasma was separated, and used for all biochemical analyses. For tissue analyses, hearts were excised, weighed, and divided to enable parallel molecular and histological assessments. Three hearts per group (n = 3/group) were snap-frozen in liquid nitrogen and stored at −80 °C for western blotting and molecular assays, while the remaining three hearts per group (n = 3/group) were fixed in 4% paraformaldehyde for histological and immunohistochemistry studies. 

The Institutional Animal Ethics Committee of ICMR-NIN approved all animal experimentation procedures and protocols (IAEC Approval No: ICMR-NIN/IAEC/2022-I/002). The animal study was conducted in accordance with the Committee for Control and Supervision of Experiments on Animals (CCSEA) guidelines.

### 4.3. Oral Glucose Tolerance Test (OGTT) and Homeostatic Model of Insulin Resistance (HOMA-IR)

OGTT was performed at the end of the experiment to assess glucose metabolism. Rats were fasted for 12 h, and fasting blood glucose (FBG) levels were measured from the tail vein using a glucometer (Accu-Chek, Roche Diagnostics, Mannheim, Germany). An oral glucose load of 2 g/kg body weight was administered via oral gavage, and blood glucose levels were measured at 0, 30, 60, and 120 min. By monitoring variations in blood glucose levels, glucose clearance was evaluated, and the area under the curve (AUC) was calculated. HOMA-IR was used to measure insulin resistance using the following formula: [fasting insulin (ng/mL) × fasting glucose (mmol/L) × 18]/22.5.

### 4.4. Assessment of Whole-Body Composition by Dual-Energy X-Ray Absorptiometry (DEXA)

Whole-body composition was assessed at the end of the experimental period using dual-energy X-ray absorptiometry (DEXA; QDR Discovery A, S/N 82382, Hologic, Waltham, MA, USA). Animals were anesthetized with a combination of ketamine/xylazine and subjected to a 2–3 min scan. The DEXA analysis provided measurements of total body mass, lean body mass, fat mass, percentage lean body mass (%LBM), and percentage body fat (%BF), which were used to evaluate changes in body composition associated with the experimental interventions.

### 4.5. Biochemical Analysis

Blood samples were collected from the retro-orbital plexus into EDTA-coated tubes and centrifuged at 3000 rpm for 20 min at 4 °C. Plasma was separated and stored at −80 °C until further analysis. Plasma insulin levels and carboxymethyl lysine (CML) levels were measured using commercially available ELISA kits. Plasma lipid parameters, including triglycerides and total cholesterol, were estimated using commercially available enzymatic assay kits from Biosystems according to the manufacturer’s instructions. Plasma protein carbonyls, a marker of oxidative protein damage, were determined by their reactivity with 2,4-dinitrophenylhydrazine [36]. Protein carbonyl groups react with DNPH to form 2,4-dinitrophenylhydrazone (DNP-hydrazone) derivatives, which are measured and quantified spectrophotometrically at 365 nm. Plasma lipid peroxidation was assessed using the thiobarbituric acid-reactive substances (TBARS) assay [36]. This assay quantifies malondialdehyde (MDA), a major end product of lipid peroxidation resulting from oxidative degradation of polyunsaturated fatty acids. MDA reacts with 2-thiobarbituric acid (TBA) under high temperature and acidic conditions to form a pink-colored MDA-TBA adduct (trimethine complex), which is measured spectrophotometrically at 533 nm.

The plasma AGE index (AGI) was determined by measuring Maillard-specific fluorescence after sequential dilution of plasma in the buffer, and linear regression was performed to obtain slope values, termed AGI, in arbitrary units. The AGE fluorescence was measured using a fluorescence spectrophotometer (Jasco-FP-6500, Tokyo, Japan) with emission/excitation spectra at 370/440 nm [36]. A total of 100 µg/mL of heart tissue homogenate was measured for non-tryptophan fluorescence by exciting the samples at 370 nm and recording the emission at 440 nm using a fluorescence spectrophotometer (Jasco FP-6500, Tokyo, Japan) [37].

### 4.6. Histological Staining

Cardiac tissues were fixed in formaldehyde, processed, paraffin-embedded, and sectioned at approximately 4 μm. Tissue sections were mounted on glass slides and stained with hematoxylin and eosin (H&E) to assess myocardial morphology. Masson’s Trichrome staining was performed to evaluate collagen deposition and myocardial fibrosis. Stained sections were examined and imaged using a light microscope (Olympus, IX73, Tokyo, Japan), and the images were quantified using FIJI/ImageJ 1.54p software (NIH, Bethesda, MD, USA).

### 4.7. Immunoblotting

Heart tissue homogenate was prepared using RIPA lysis buffer (pH 7.4) containing protease and phosphatase inhibitors. The homogenate was centrifuged at 12,000 rpm for 30 min at 4 °C. The supernatant was collected, and protein estimation was performed using Lowry’s method. Equal amounts of protein were loaded and resolved by 12% sodium dodecyl sulfate-polyacrylamide gel electrophoresis (SDS-PAGE) and transferred onto a nitrocellulose membrane. Blocking was performed for one hour with 5% skimmed milk prepared in Tris-buffered saline (TBS, pH 7.4), and later the blots were incubated overnight with the respective primary antibodies (CML, MGO, NF-κB, p-NF-κB) at 4 °C. After incubation, the membranes were washed using TBS with Tween 20 (TBST) and incubated with corresponding secondary antibodies. Immunoblots were developed using an enhanced chemiluminescence substrate (Bio-Rad, Hercules, CA, USA), and images were captured (iBright^TM^ FL 1500, Thermo Scientific, Waltham, MA, USA). Band intensities were quantified using FIJI/ImageJ 1.54p software (NIH, Bethesda, MD, USA), and protein expression was normalized to the internal control, GAPDH.

### 4.8. Immunohistochemistry

The formalin-fixed heart tissues mounted on gelatin-coated slides were deparaffinized by heating at 65 °C for 1 h and incubated in xylene for 10 min twice. The sections were hydrated with decreasing ethanol concentrations (90%, 75%, and 50%), followed by distilled water, 10 min at each step. The deparaffinized sections were heated in 0.1 M citrate buffer (pH 6) three times, 4 min each, for antigen retrieval. Later, blocking was performed using 3% goat serum in PBS for 1 h. After blocking, sections were incubated with the respective primary antibodies (fibronectin, Col1α, α-SMA, and RAGE), diluted in PBS with 1.5% goat serum, overnight at 4 °C. The following day, sections were incubated at room temperature for 1 h, washed with phosphate-buffered saline with Tween-20 (PBST) three times, 10 min each, and incubated with Alexa Fluor-conjugated secondary antibodies diluted in PBS containing 1.5% goat serum for 2 h at room temperature. The sections were mounted using 4′,6-diamidino-2-phenylindole (DAPI) as a nuclear counterstain, and the slides were imaged using a fluorescence microscope (Leica Microsystems and Zeiss, Wetzlar, Germany). The images were quantified using FIJI/ImageJ software (NIH, Bethesda, MD, USA).

### 4.9. Statistical Analysis

Statistical analysis was performed using GraphPad Prism 8.0 (GraphPad Software, San Diego, CA, USA). Data were reported as mean ± SD. Statistical analysis was performed using two-way ANOVA to evaluate the effects of diet (control vs. HFHS) and treatment (EA vs. no EA), followed by Tukey’s post hoc test for multiple comparisons. A *p*-value of less than 0.05 (*p* ˂ 0.05) was considered statistically significant. Significant differences are indicated as * *p* ˂ 0.05, ** *p* ˂ 0.01, *** *p* ˂ 0.001, **** *p* ˂ 0.0001.

## 5. Conclusions

Overall, the findings of the present study suggest that prolonged HFHS feeding induces metabolic dysfunction accompanied by cardiac remodeling through AGE-RAGE signaling and activates markers for oxidative damage. EA supplementation exerts protective effects by improving metabolic parameters, reducing AGE accumulation and RAGE expression, and attenuating myocardial fibrosis, suggesting a modulatory effect on these interconnected pathways. These results support EA’s potential as a dietary compound in attenuating HFHS-diet-induced metabolic dysfunction.

### Limitations

This study did not include hemodynamic parameters, experiments on the antioxidant defense system, equivalent/translational doses of EA, or detailed cardiac functional assessments, such as echocardiography, which would have strengthened the evaluation of cardiac performance. Long-term translational studies and clinical investigations will also be needed to establish EA’s therapeutic potential for prevention and management. Future research can incorporate both sexes to assess possible differences in how each sex responds to HFHS diets and ellagic acid supplementation.

## Figures and Tables

**Figure 1:**
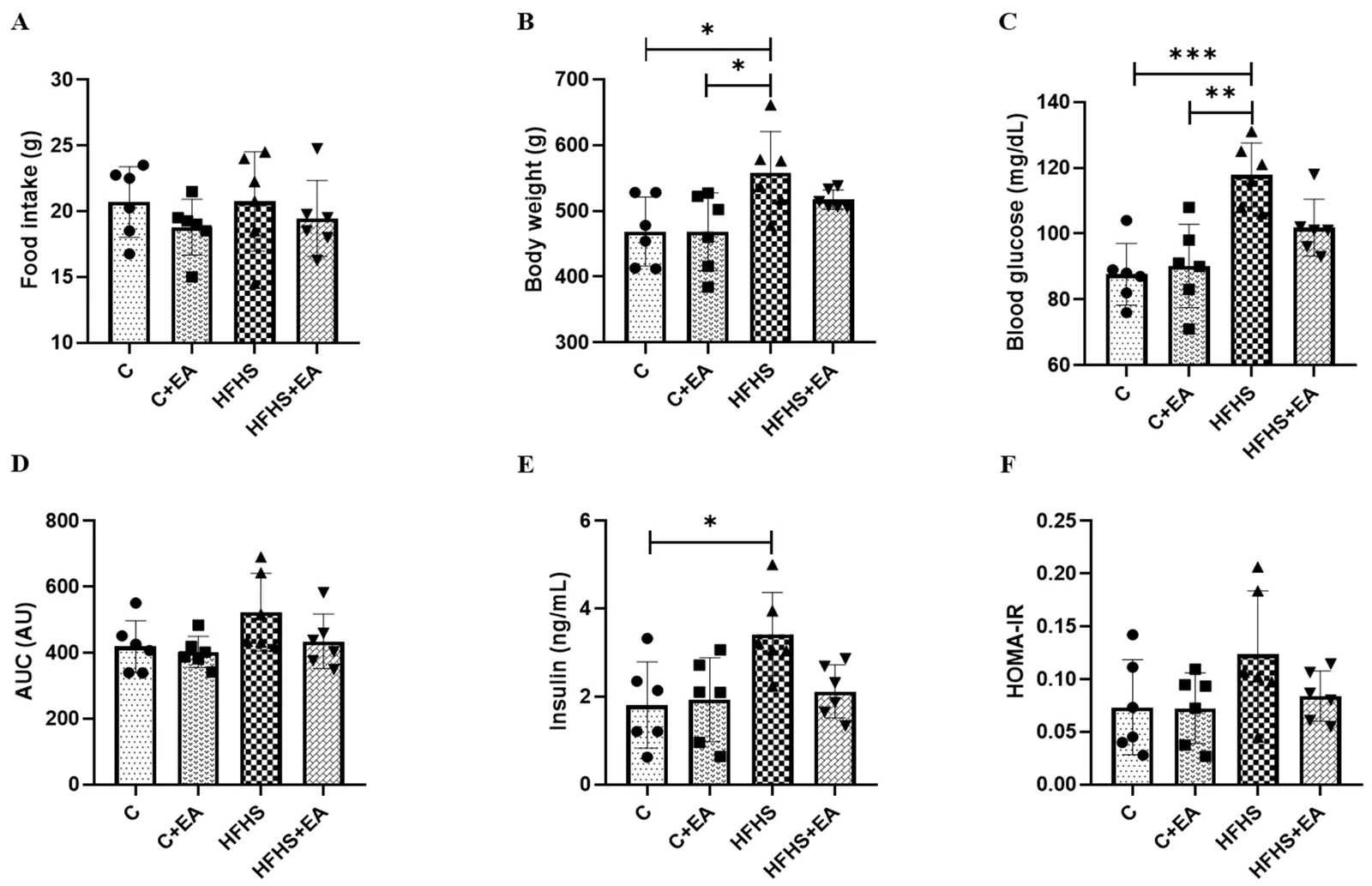
Effect of ellagic acid on metabolic parameters. (**A**) Food intake. (**B**) Body weights. (**C**) Fasting blood glucose levels. (**D**) Area under the curve calculated from OGTT. (**E**) Fasting plasma insulin levels. (**F**) Insulin resistance index calculated using HOMA-IR. [C = control diet; C+EA = control diet supplemented with 2% ellagic acid; HFHS = diet containing high-fat, high-sucrose; and HFHS+EA = HFHS diet supplemented with 2% ellagic acid]. All data were analyzed using two-way ANOVA and Tukey’s test. Data are presented as mean ± SD (n = 6). Significant differences are indicated as * *p* ˂ 0.05, ** *p* ˂ 0.01, *** *p* ˂ 0.001.

**Figure 2:**
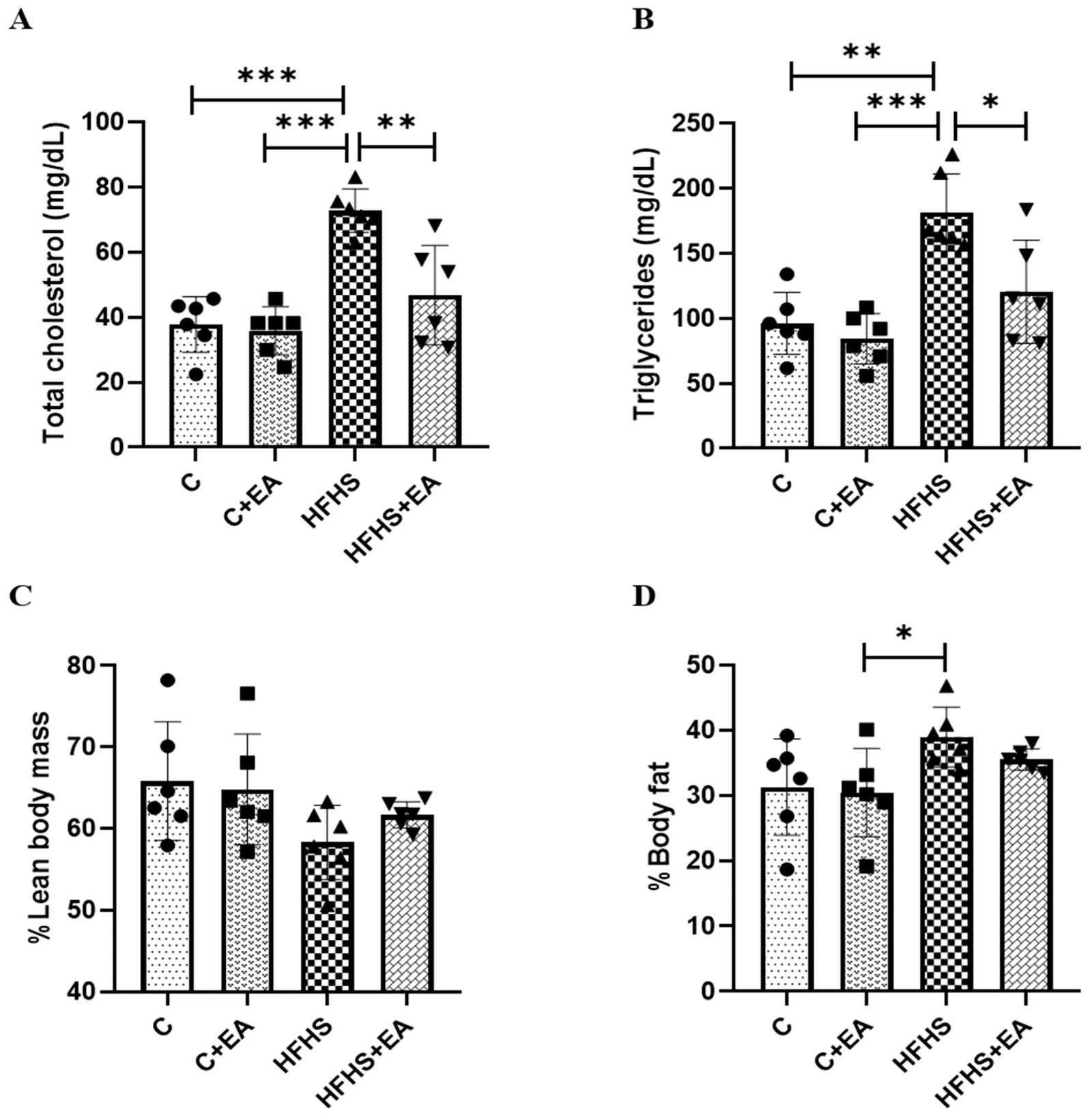
Effect of ellagic acid supplementation on lipid profile and body composition. (**A**) Plasma total cholesterol. (**B**) Plasma triglycerides. (**C**) Percentage lean body mass. (**D**) Percentage body fat. [C = control diet; C+EA = control diet supplemented with 2% ellagic acid; HFHS = diet containing high-fat, high-sucrose; and HFHS+EA = HFHS diet supplemented with 2% ellagic acid]. All data were analyzed using two-way ANOVA followed by Tukey’s test. Data are expressed as mean ± SD (n = 6). Significant differences are indicated as * *p* ˂ 0.05, ** *p* ˂ 0.01, *** *p* ˂ 0.001.

**Figure 3:**
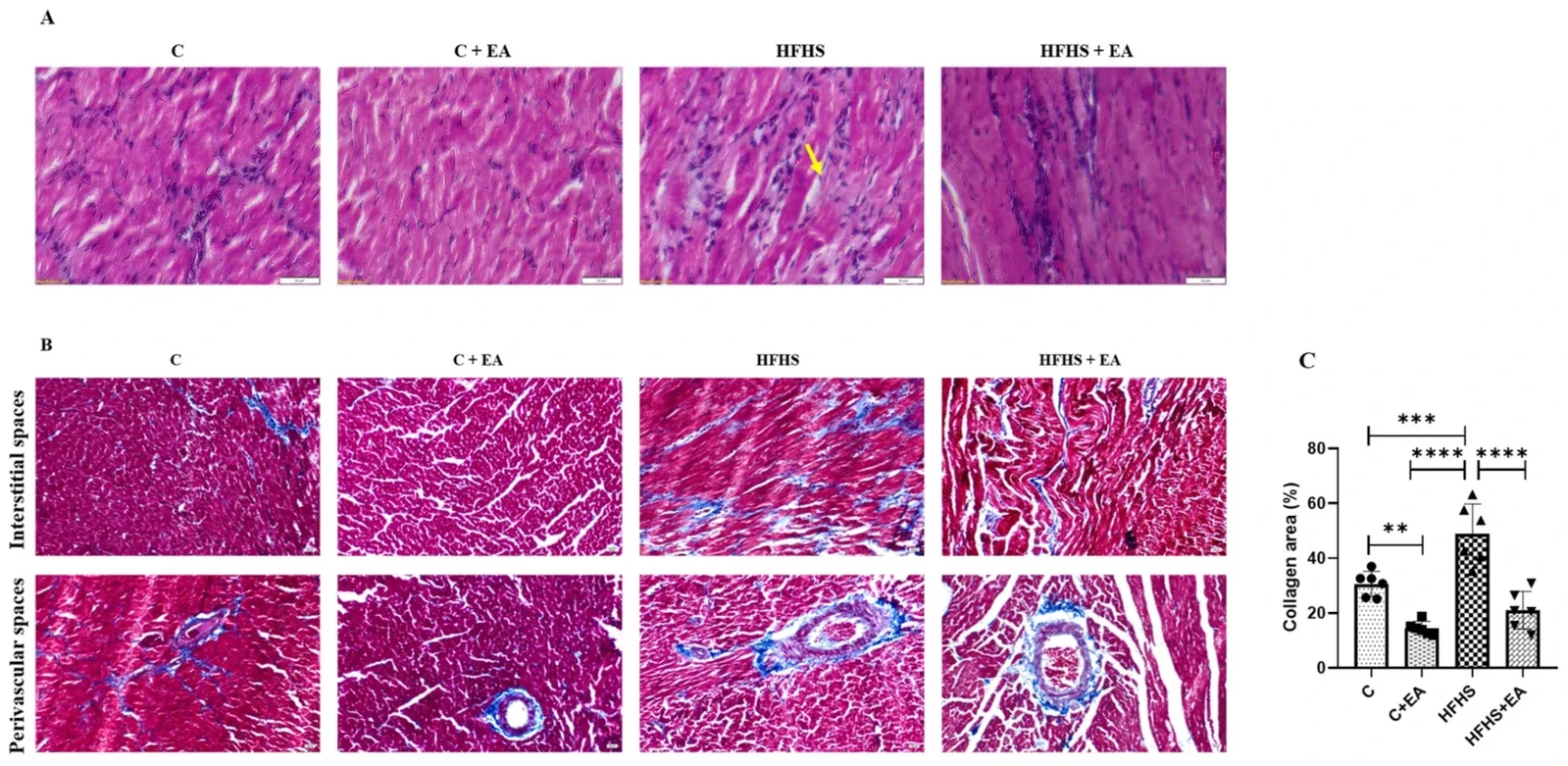
Ellagic acid prevents morphological changes in the heart. (**A**) Hematoxylin and eosin staining of heart sections (yellow arrow indicates disorganization of myocardial fibers). Magnification 400×, scale bar = 50 µm. (**B**) Masson’s Trichrome staining of heart sections, showing collagen deposition (nuclei are stained black, cytoplasm and muscle fibers are stained red, and collagen is stained blue). Magnification 200×, scale bar = 25 µm. (**C**) Percentage of collagen area calculated from MT stain images. [C = control diet; C+EA = control diet supplemented with 2% ellagic acid; HFHS = diet containing high-fat, high-sucrose; and HFHS+EA = HFHS diet supplemented with 2% ellagic acid]. All the data were analyzed using two-way ANOVA followed by Tukey’s test. Data are expressed as mean ± SD (n = 3). Significant differences are indicated as ** *p* ˂ 0.01, *** *p* ˂ 0.001, **** *p* ˂ 0.0001.

**Figure 4:**
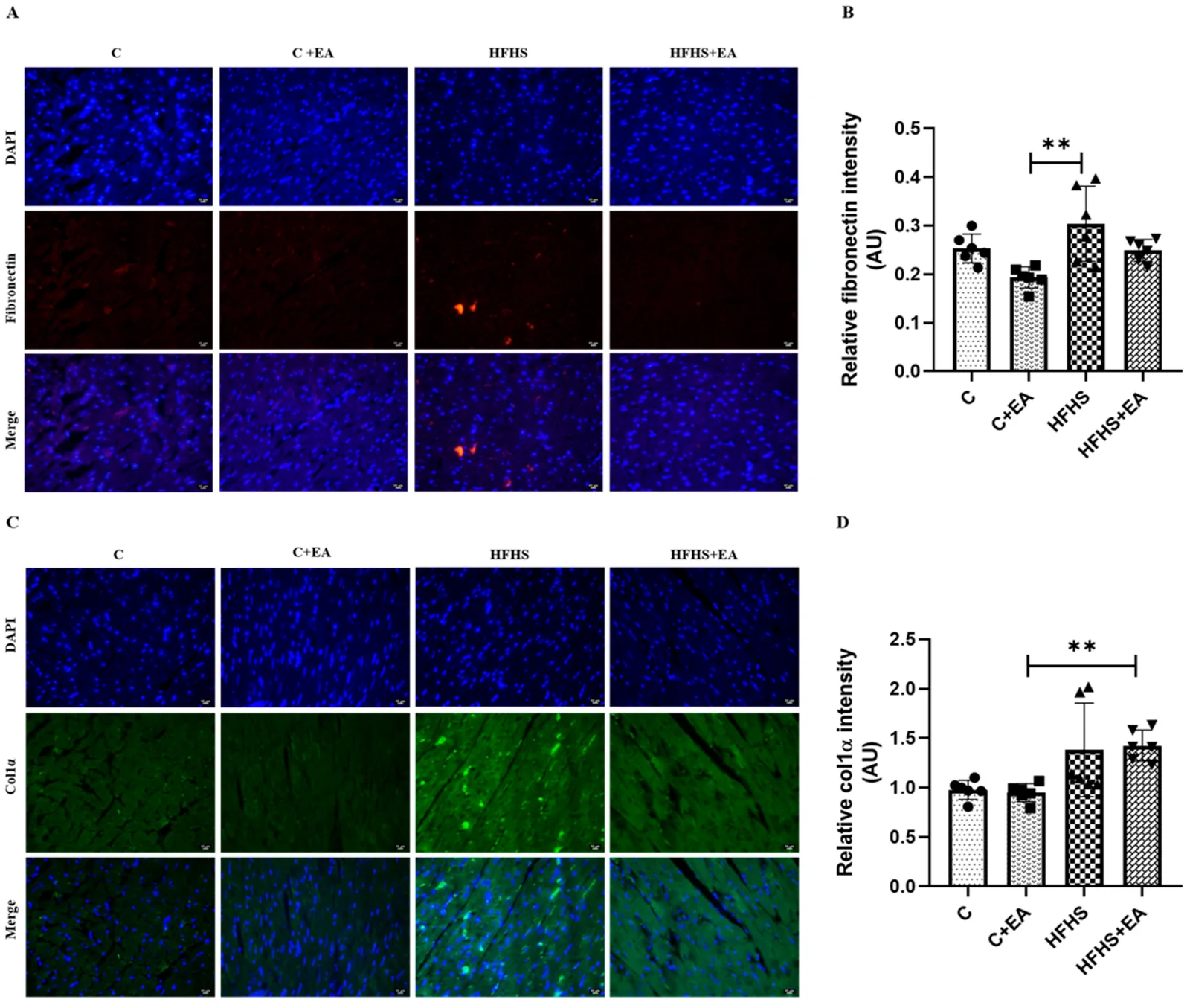
(**A**)—Representative image of immunofluorescence staining of fibronectin in the heart. (**B**)—Relative fluorescence intensity of fibronectin. (**C**)—Representative image of immunofluorescence staining of Col1α in the heart. (**D**)—Relative fluorescence intensity of Col1α. Blue: Nuclear stain with DAPI. Red: Fibronectin stain. Green: Col1α. 400× magnification, scale bar = 10 µm. [C = control diet; C+EA = control diet supplemented with 2% ellagic acid; HFHS = high-fat, high-sucrose diet; and HFHS+EA = HFHS diet supplemented with 2% ellagic acid]. All the data were analyzed using two-way ANOVA followed by Tukey’s test. Data are expressed as mean ± SD (n = 3). Significant differences are indicated as ** *p* ˂ 0.01.

**Figure 5:**
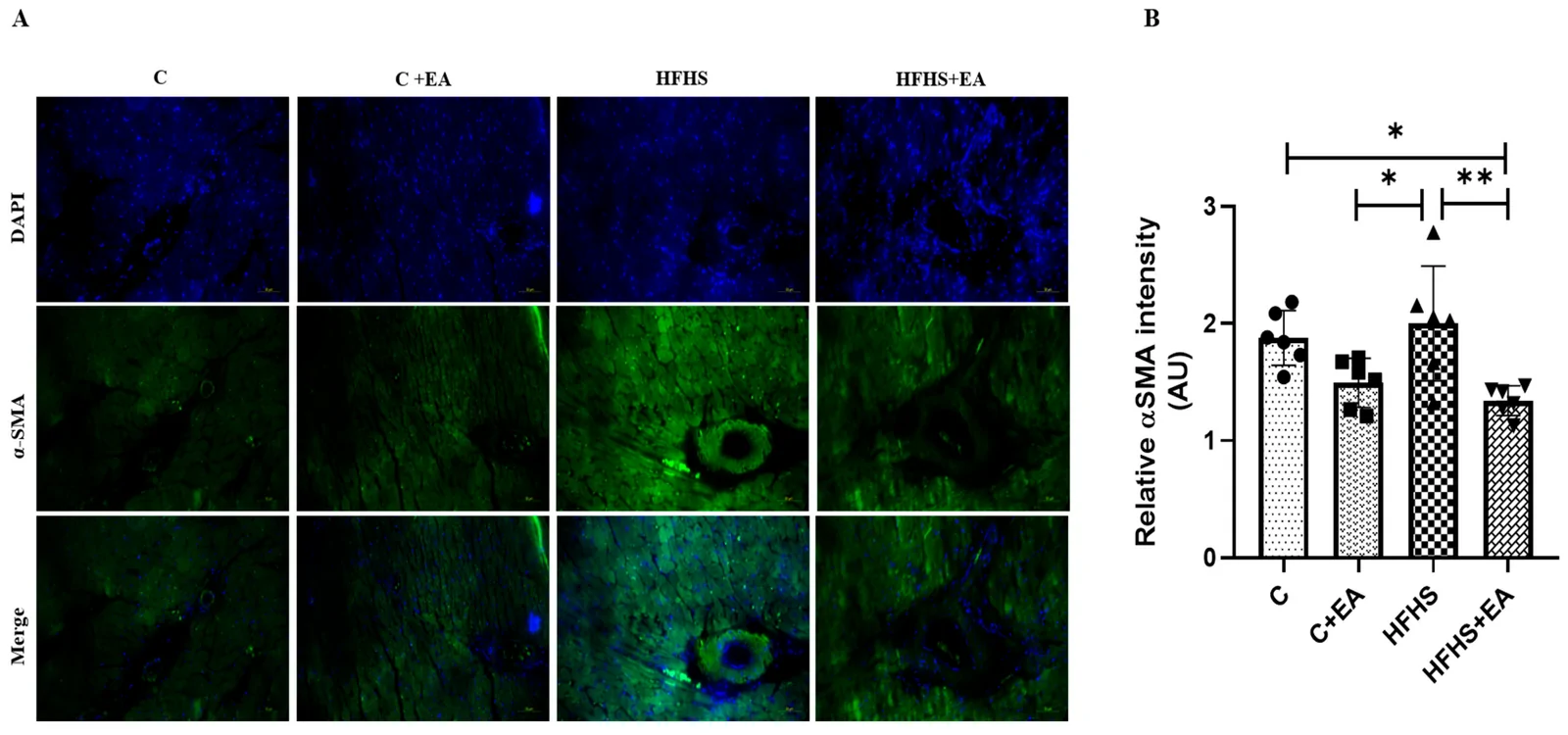
(**A**)—Representative image of immunofluorescence staining of α-SMA in the heart. (**B**)—Relative fluorescence intensity of α-SMA. Blue: Nuclear stain with DAPI. Green: α-SMA. 400× magnification, scale bar = 50 µm. [C = control diet; C+EA = control diet supplemented with 2% ellagic acid; HFHS = high-fat, high-sucrose diet; and HFHS+EA = HFHS diet supplemented with 2% ellagic acid]. All the data were analyzed using two-way ANOVA followed by Tukey’s test. Data are expressed as mean ± SD (n = 3). Significant differences are indicated as * *p* ˂ 0.05, ** *p* ˂ 0.01.

**Figure 6:**
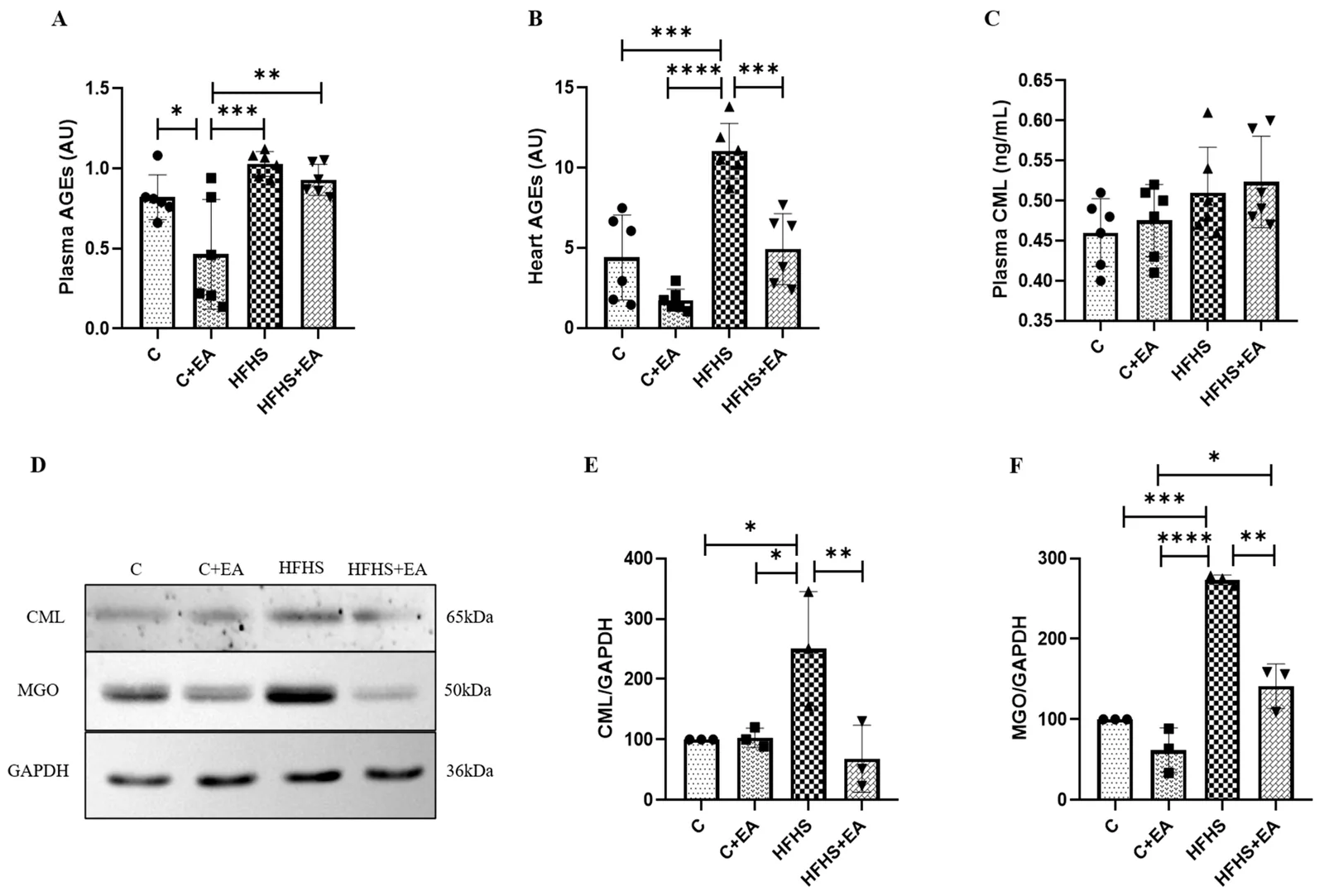
Ellagic acid supplementation inhibits AGE accumulation. (**A**)—Plasma non-tryptophan fluorescence of AGEs (n = 6). (**B**)—Fluorescence of AGEs in heart (n = 3). (**C**)—Plasma CML (n = 6). (**D**)—Representative immunoblots of CML and MGO (n = 3). (**E**,**F**)—Quantification of CML and MGO normalized to GAPDH (n = 3). [C = control diet; C+EA = control diet supplemented with 2% ellagic acid; HFHS = diet containing high-fat, high-sucrose; and HFHS+EA = HFHS diet supplemented with 2% ellagic acid]. Data are expressed as mean ± SD and analyzed by two-way ANOVA followed by Tukey’s test. Significant differences are indicated as * *p* ˂ 0.05, ** *p* ˂ 0.01, *** *p* ˂ 0.001, **** *p* ˂ 0.0001.

**Figure 7:**
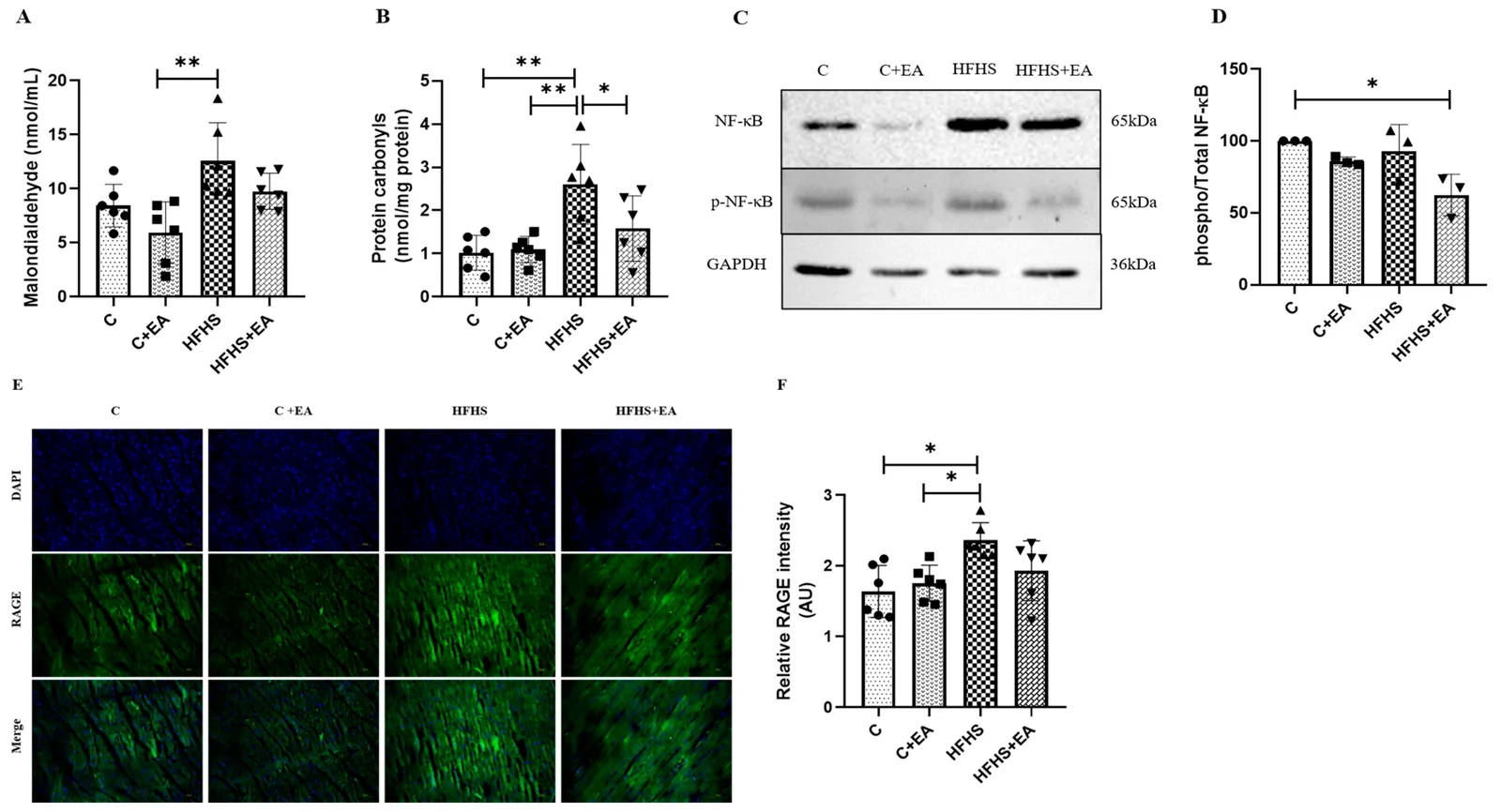
Ellagic acid supplementation reduced oxidative stress and inflammation in HFHS diet-fed rats. (**A**)—Plasma malondialdehyde levels (n = 6). (**B**)—Plasma protein carbonyls (n = 6). (**C**)—Representative immunoblots showing the expression of NF-κB and p-NF-κB in the heart (n = 3). (**D**)—Quantitative analysis of p-NF-κB normalized to Total NF-κB (n = 3). (**E**)—Representative image of immunofluorescence staining of RAGE in the heart. Blue: Nuclear stain with DAPI. Green: RAGE. Merged: DAPI and RAGE. 400× magnification, scale bar = 10 µm (n = 3/group). (**F**)—Relative fluorescence intensity of RAGE. [C = control diet; C+EA = control diet supplemented with 2% ellagic acid; HFHS = high-fat, high-sucrose diet; and HFHS+EA = HFHS diet supplemented with 2% ellagic acid]. Data are expressed as mean ± SD and analyzed using two-way ANOVA followed by Tukey’s test. Significant differences are indicated as * *p* ˂ 0.05, ** *p* ˂ 0.01.

## Data Availability

The original contributions presented in this study are included in the article. Further inquiries can be directed to the corresponding author.

## Abbreviations

The following abbreviations are used in this manuscript:

HFHS	High-fat and high-sucrose
CF	Cardiac fibrosis
AGEs	Advanced glycation end products
EA	Ellagic acid
CVDs	Cardiovascular diseases
RAGE	Receptor for AGEs
ECM	Extracellular matrix
ASEAN	Association of Southeast Asian Nations
OGTT	Oral glucose tolerance test
FBG	Fasting blood glucose
AUC	Area under the curve
HOMA-IR	Homeostatic model of insulin resistance
DEXA	Dual-Energy X-ray Absorptiometry
Col1α	collagen type I alpha
α-SMA	α-smooth muscle actin
CML	Nε-(carboxymethyl)lysine
MGO	Methylglyoxal
MDA	Malondialdehyde
NF-κB	nuclear factor kappa-light-chain-enhancer of activated B cells
p-NF-κB	Phospho NF-κB
DAPI	4′,6-diamidino-2-phenylindole
